# The Field and Function of the Negro College in STEM 2025

**DOI:** 10.1093/icb/icaf105

**Published:** 2025-06-19

**Authors:** Joseph L Graves

**Affiliations:** MacKenzie Scott Endowed Professor of Biology, North Carolina A&T State University, Greensboro, NC 27411, USA

## Abstract

African Americans are still underrepresented in science, technology, engineering, and math (STEM) careers. This fact has had profoundly negative impacts on both the lives and fortunes of African Americans, as well as our society as a whole. This pattern of underrepresentation does not result from an inherent mental deficiency or lack of interest in science by African Americans. Rather, this results from historical and ongoing discrimination against this group throughout American society. This paper recounts this discrimination in the context of society as a whole and higher education with a specific emphasis on STEM careers in biology and organismal biology in particular. It offers concrete recommendations for changing the enterprise of science such that it can foster greater inclusion of African Americans via the support for and expansion of the capacity of Historically Black Universities and Colleges (HBCUs).

## Introduction: those who forget their history are doomed to repeat it

“Unless the American Negro today, led by trained university men of broad vision, sits down to work out by economics and mathematics, by physics and chemistry, by history and sociology, exactly how and where he is to earn a living and how he is to establish a reasonable life in the United States or elsewhere, unless this is done the university has missed its field and function and the American Negro is doomed to be a suppressed and inferior caste in the United States for incalculable time.”

W.E.B. Du Bois, *The Field and Function of the Negro College*, 1933.

William Edward Burghardt Du Bois was the first African American to graduate from Harvard University (1895). While his formal degree was in history, much of his career was spent explaining the impacts of structural racism on African Americans and American society as a whole. His “Field and Function” essay of 1933 was directed to the leadership of the Negro colleges in the South. (All quotations in this article are provided as originally written. The term “Negro” was derived from the Spanish word for black. It was the preferred term used by African Americans scholars to describe persons of African descent in this period. DuBois did not [nor did any other scholars of this time period] use gender neutral language. However, when DuBois spoke of men in this passage, he also meant women, per the comments of Herbert Aptheker pg. xi in the introduction of *The Education of Black People*, and his most recent biographer David Levering Lewis). During Jim Crow racial segregation, higher education for African Americans was conducted primarily under the “trade school” model, which was advocated by Booker T. Washington in the early 20th century (Lewis 2014). While Du Bois understood the necessity of such training opportunities for the newly freed African American population, he felt that this could and should not be the only emphasis for African American higher education (DuBois 1973). Specifically, he felt that Negro colleges should offer a liberal arts education, which must also include training in mathematics and science. However, during the 20th century these institutions failed to achieve the mission of producing the scholars required to create economic, social, and political freedom for African Americans. Yet, this failure cannot be laid at the feet of Negro college leadership. No strategy they could have deployed would have made a difference. The suppression of African American achievement was intentional and systematic. It occurred because European American society (particularly in the former Confederate states) never wanted African Americans or their institutions to succeed in achieving social equality (and I argue that this is still the case today). This does not mean that all European Americans are racists, indeed many have taken an active role in the fight against structural racism. I would not be writing this essay if several of them had not served as my mentor during my training in evolutionary biology (Graves 2022). However, historically the majority of European Americans have failed to act against or have bought into a social/political system that has and still is systematically denying opportunity to African Americans (Lipsitz 2005). Most do not realize that they support this system because they do not understand how it operates. For example, the most commonly used and simple definition of racism is prejudice plus power (Graves and Goodman 2022). Even well-meaning persons who reside at the top of systems of racial hierarchy tend to focus on individuals acts of racism. They will often claim that racism no longer exists because their colleagues and friends do not display racist behavior in public. However, during my years of graduate training in the 1980’s, I was called “nigger” so much that it could have been my middle name. Thus, personal or individual racism is important because without it, the second and most important form of racism—institutional and structural racism—would lose ideological support.

For most of the 20th century, the majority of European Americans accepted the inferiority of Africans as scientific and socially agreed “fact” (Graves 2025a). For example, much of the rancor against the teaching of evolution in the early 20th century was due to the logical implications of Darwin’s descent with modification dictum that established the kinship of Africans and Europeans, thus eroding the superiority of the latter (DuBois 1997; Wineapple 2024). Indeed, serious scientific challenges against the theories of Negro racial inferiority did not appear until mid-century (Montagu 1942; Graves 2023). Yet, despite this work, biological anthropologists such as Carleton Coon argued against school integration in the 1960’s due to the danger of racial amalgamation (Jackson 2001). Nobel prize winning scientists and professors at America’s premier universities argued (and some still do) for the intellectual inferiority of African Americans utilizing a pseudoscience masquerading as an evolutionary genetic basis into the 1990’s (Chase 1980; Herrnstein and Murray 1994; Rushton 1995).

The combination of scientific support and social belief in the inferiority of African Americans allowed and still allows powerful institutions to discriminate against them. Some argue that the fact of African American inferiority is supported by their condition in American society. In virtually, every measure of social position and well-being (e.g., life expectancy, infant mortality, educational attainment, and wealth) African Americans are at or near the bottom. This has been true across American history, such much so that in 1897 Frederick Hoffman predicted that Negroes would soon go extinct due to their inability to compete in free society (Hoffman 1896). This argument is the basis of modern “color-blind” racism, which claims that structural racism no longer exists and posits that the failure of African Americans in our society is the result of their own genetic or cultural faults (Graves 2015). This is thinking is behind President Trump’s assaults on diversity, equity, and inclusion programs (Trump Presidential Executive Order, January 20, 2025). It calls DEI wasteful because it is not needed and makes the false claim that DEI programs are discriminatory. The order does not explain who is being discriminated against, but based upon Trump’s past thinking he is clearly claiming that socially defined “whites” and males are the targets of DEI discrimination.

The proponents of African American genetic inferiority have never made a scientifically valid case to support that claim. This is because the demonstration that any individual or population is genetically inferior requires that the individuals or populations in question share a common and equal environment. In my experimental work in life history evolution, we never compared the survivorship and reproduction of our experimentally evolved stocks unless they had been reared in at least two generations of a common environment (Graves et al. 2017). This of course has never been true for persons of African descent in American society.

## African American history 101

The differentiation of African and European indentured servants began as early as 1670 (Suryani 2023). The former could work off their servitude and the latter were enslaved for life throughout the slave holding colonies (and later states of the union). These states would make laws against the education of enslaved persons (Rothman 2005). The result of chattel slavery was a unidirectional flow of labor and the resources they produced from African to European-Americans. The actual amount of this transfer is a staggering, estimated at 18.5 quadrillion dollars in 2018 currency between 1776 and 1865 (Craemer et al. 2020). Furthermore, enslaved person’s bodies also served as raw material that built American medical practice via anatomical study as well as the wealth to establish the universities (Wilder 2013).

The end of slavery did not result in an end to the economic and social exploitation of African Americans. Prior to becoming president, Abraham Lincoln was convinced of both the inferiority of Africans and the impossibility of freed African Americans being integrated into American society. His views changed during his presidency in large part to the influence of people like Frederick Douglass and his own observation of the heroism and sacrifice of African Americans during the Civil War (Escott 2009). We do not know how reconstruction would have proceeded if Lincoln had lived, but his successor Andrew Johnson was a sympathizer with the former Confederate states desire to reinstitute white supremacy in the south. They managed this with brutal force, including the wanton murder of African Americans and the removal of elected African American representatives from state legislatures by force (Du Bois 1940). By 1877, all attempts to secure social and political rights for African Americans in the former confederate states, and in the words of the eminent historian of the period, Kenneth Stampp, the south was “redeemed” (Stampp 1967).

The failure of reconstruction led to the implementation of “black codes” throughout the former Confederate states. They limited social and political possibilities for African Americans via enforcement of things, such as restricting trades and occupations to European Americans, restricting the movement of African Americans by requiring passes, preventing African Americans from voting, serving on juries, or election to office. They also prevented African Americans from owning various forms of property, requiring residential segregation, and preventing African Americans from owning fire arms, thus making them vulnerable to racial terror conducted by organizations such as the Ku Klux Klan.

In addition, the philanthropy of wealthy European Americans toward African American institutions was not really to uplift “the race” toward social equality. Rather it was to ensure that Negroes would become docile and compliant workers via the trade school model that was promulgated out of Tuskegee by Booker T. Washington. Du Bois wrote about the motives in his biography *Dusk of Dawn*:

“They were capitalists and employers and yet in most cases sons, relatives, or friends of the abolitionists.. . . These younger men believed that the Negro problem could not remain a problem of philanthropy. These Negroes were not to be encouraged as voters in the new democracy, nor were they to be left at the mercy of the reactionary South. They were good laborers and they might be better.. . . Properly guided they would restrain the unbridled demands of white labor, born of the Northern labor unions and now spreading to the South.”

Du Bois was not alone in coming to this conclusion, the Reverend Dr Martin Luther King Jr also discussed this issue in his final book *Where Do We Go From Here* (King 1967).

Discrimination against African Americans did not just occur in the south. The practice of redlining is an example. By the 1930’s, it was a widespread practice in the United States to deny loans and housing to African Americans. The Home Owners Loan Corporation (HOLC) was established in 1933 as part of the New Deal programs of President Franklin Delano Roosevelt. The HOLC created color-coded maps of properties based upon the risk of lending money in specific neighborhoods. The socially defined race of the neighborhood’s inhabitants played a large role in determining the risk. Communities with large percentages of African Americans and Latinos were considered highest risk and were colored red. The practice of “redlining” led to different capacities for accumulating wealth. Today, the median wealth of European families is 10-fold that of African American families. Much of this resulting from the ability of the former to secure bank loans to own their homes, while the latter group could not (Aaronson et al. 2021).

After World War II ended, a series of civil rights related executive orders, court decisions, and legislation began to erode white supremacy in the United States. These included President Truman’s executive order to integrate the military in 1948, the Supreme Court’s decision to strike down segregation in education in Brown v. Board of Education 1954, the Civil Rights Act of 1964, and the Voting Rights Act of 1965. However, all of these actions were piece meal in their implementation due to significant and ongoing resistance against them by large sectors of the European American population. Indeed, in 1968, the Republican Party rebranded itself as the party of “white people” to capitalize on this racial resentment (Mayer 2002). Nixon’s 1972 victory was aided by white resentment to busing to achieve school desegregation. The Ford administration of 1976 gave up on any policies to achieve racial equality. Reagan opposed virtually every civil rights bill ever proposed. Bush utilized white outrage over the “Willie Horton” incident to defeat Michael Dukakis. Clinton’s 1996 campaign succeeded because he was willing to curtail affirmative action policies (a term and policy that had originated with the Kennedy administration). In 2023, affirmative action was struck down by the Trump appointed Supreme Court. In 2025, among Trump’s first executive orders was the dismantling of all federal diversity, equity, and inclusion programs (Trump 2025). American history, as viewed from the perspective of African Americans can be summarized as follows: 1619–1865: chattel slavery; 1865–1964: second class citizenship by law; 1965–2024: inconsistent federal government attempts to advance racial justice; and 2025–? repudiation of racial justice by the Trump administration. Over the course of my career, I have found that few persons of European descent (and even fewer of those who are scientists) are familiar with the details of African American history. Understanding that history is critical to appreciate the argument I make below concerning the importance in Historically Black Universities and Colleges (HBCUs) in science, technology, engineering, and math (STEM) research and training.

## A brief history of African American higher education

I have already written a paper that reviews the history of African American higher education in the context of evolutionary science (Graves 2019). In summary, that paper provides definitions of the various subpopulations within the African diaspora. The term African American refers to individuals who are the descendants of persons who were enslaved in the United States. It goes on to describe the African American experience in higher education. In short, during chattel slavery (1776–1865), education for African Americans was discouraged (and in many places illegal). In 1862, American higher education received a major boost by the passage of the Morrill Land Grant Act (Duemer 2007). This established 57 universities in 37 states. However, after the Civil War ended, land grant institutions in the former Confederate states refused entry to persons of African descent. Very few African Americans were admitted to higher education in the former union states as well. For this reason, congress authorized a second Morrill Land Grant Act of 1890 (Neyland and Fahm 1990). In comparison, the 1890 act, only established 19 universities in 18 states.

The 1890 Morrill Act was passed by the US Congress in the same year that the state of Louisiana passed the “Separate Car Act (Medley 2012).” This act was designed to separate transportation accommodations by socially defined race. Upon hearing of the law, the non-white population of Louisiana planned to challenge it. Homer Plessy had ridden the street cars of New Orleans with no interference during Reconstruction. By ancestry Plessy was 7/8th European and 1/8th African (classified by the state as an “Octoroon.”) Plessy was arrested for riding in the Whites only section in 1892. The case may it way to the Supreme Court of the United States in 1896. The decision against Plessy (Plessy v. Ferguson, SCOTUS, 1896) meant that states could now legally segregate all public facilities by race, including public schools and universities. The proviso, for this separation was that funding for these facilities must be “equal.” This never happened, in fact, state legislatures in throughout the South routinely pilfered funds that were earmarked for Black, to pay for White education (Harris 2021).

## On going racial inequality and injustice in American education

Throughout the 20th century, and now in the 21st century funding for education at all levels has differed by socially defined race (Kozol 2024). Over the 20th century residential segregation maintained a system of underfunding public schools by socially defined race (Ryan 1999). The American public school funding system varies between state, local, and federal funding. Public schools in the South (where the majority of African Americans live) spend less money per student than other regions (Morgan 2022). However, just knowing the amount of money spent per student does not tell the whole story. Children living in wealthier districts are also raised by parents who have higher levels of education and also attend schools with better infrastructure. This has resulted in an achievement (or opportunity) gap between African and European Americans. Classical studies suggest that this gap is the result of the combination of socioeconomic and cultural factors (Paige and Witty 2010). However, the interaction of these is complex. For example, the relationship between parent’s educational level and the achievement of their children is not consistent. Yet there are examples, of how in urban schools, parental education and involvement showed significant effects reducing the high school drop-out rate and the overall highest grade achieved by the students (Barnard 2004; Park and Holloway 2016; Assari and Caldwell 2019; Assari et al. 2021). However, there is evidence that the effect of parental education and socioeconomic status operates differently for African Americans than it does European Americans. In the case of the former, one recent study showed diminishing returns, but no such effect was seen for the latter (Assari 2019).

The inequities in school funding by socially race are getting worse, not better. In 2022, the Education Trust reported that school districts who serve majority minority students receive considerably less funding from state and federal sources, as much as $2700 dollars per student (Education Trust 2022). This amount of differential funding on average would allow these districts to hire at least three more teachers, provide tutoring for students who need additional help, or to buy a laptop for each student. In addition, throughout the former Confederate states, voucher systems are allowing the establishment of segregation academies. Case in point, the state of North Carolina recently passed a law providing vouchers without regard to parental income to allow more children to attend private schools (Keung Hui 2024). These programs have been shown to increase school segregation by socially defined race and class (Mickelson et al. 2008).

As a result of the ongoing inequity in school funding, HBCUs admit a disproportionate share of students from struggling school districts compared to their historically white institution (HWI) peers. Yet, state appropriations to HWIs are disproportionately larger than HBCUs within the former Confederate states. For example, in 2023–2024, the ratio of state appropriations to North Carolina State University (1860 land grant) compared to its undergraduate enrollment was 2.81, but it was only 1.81 for North Carolina A&T State University (1890 land grant, University of North Carolina 2023). This situation is repeated across the former Confederate states, as documented in the Century Foundation report on the status of HBCUs (Smith 2023). Florida, Louisiana, North Carolina, Tennessee, and Texas underfunded HBCUs in the amounts of billions, according to a letter to those states governors from Miguel Cardona, Secretary of Education and Thomas J. Vilsack, Secretary of Agriculture sent in September 2023. My institution, North Carolina A&T State University has a $2 billion funding disparity, compared with North Carolina State University at Raleigh, the original Morrill Act of 1862 land grant institution, the letter said (Figueroa 2023).

The combination of federal and state underfunding and lack of private investment means that the endowments of HBCU’s lag seriously behind that of their HWI counterparts. Table 1 lists the endowments of America’s top HWIs compared to its HBCUs. This historic underfunding of HBCUs results in an environment in which their faculty must teach disproportionately higher class loads (with students who often have various holes in their college preparation), advise larger numbers of students, and teach and conduct research in facilities that are ill-suited for 21st century STEM instruction and research. HBCU land-grant universities operate on shoestring budgets, with fewer resources for research, technology, academic instruction, student support, and community programs. Between fiscal years 2011 and 2022 alone, Black land-grant universities lost nearly $200 million in resources because states declined to provide matching funds while they fully funded their white land-grant universities. The US Department of Agriculture continues to look the other way while states engage in discriminatory and inequitable funding of the 1890 institutions. This situation has led to historical differences in research conducted at HWIs compared to HBCUs. In fiscal year 2022, HBCUs received only 658.4 million in research funding from all federal agencies compared to 44.6 billion to HWIs. In fiscal year 2021, Stanford University alone received 1.8 billion in federal research dollars. The following year, the top 15 HBCUs only received 431.4 million in research funding (National Center for Science and Engineering Statistics 2025).

**Table 1:** The endowments of the top five HWIs compared to the top five HBCUs is shown in billions of dollars. The HWI endowments are >96 times that of the HBCUs. This data was generated from a Wikipedia search of university endowments based upon data from 2023

HWIs	Billions	HBCUs	Billions
Harvard University	50.70	Howard University	0.87
Yale University	41.30	Spelman College	0.46
Stanford University	36.50	Hampton University	0.29
Princeton University	37.70	Morehouse College	0.19
Massachusetts Institute of Technology	24.60	Meharry Medical College	0.16
Total	190.8		1.97

Due to budgetary short falls, administrators at HBCUs often find themselves having to delay the purchase of new or repair to much needed infrastructure. For example, in January 2024, NCATSU had to house students in hotels (or send them home) because of the failure of its central heating plant (Fox News 2023). Our campus lags behind the R1 campuses within the UNC system with regards to modern research space. On one hand we have new construction such as the Harold Martin Engineering Research and Innovation Complex (https://www.ncat.edu/coe/martin-complex/harold-martin-complex.php). We also share the Joint School of Nanoscience and Nanoengineering facility (https://jsnn.ncat.uncg.edu/; which has state-of-the-art nanofabrication and nano-characterization instrumentation.) However, these are the exception and not the rule. The Biology department (which has the highest number of undergraduate majors on campus >900) operates out of facilities that were built in the 1960’s. They were constructed for teaching, not research. These buildings suffer from old plumbing and air handling systems such that in the winter students are often working in labs in coats, hats, and gloves; while in the summer temperatures exceed the operational thresholds of our incubators and other scientific instrumentation. None of our colleagues at the UNC Chapel Hill, UNC Charlotte, or NC State have to work under these conditions.

Underfunding also means that we do not pay the most competitive salaries for faculty or staff. For example, a comparison of full professor salaries at UNC-CH, NCSU, and NCATSU shows that NCATSU faculty get paid much less. By law, faculty salaries in the UNC system are publicly available. There are presently five full professors in the biology department at NCATSU, all hold externally funded research grants, and are well respected scientists in their fields (three are men, and two are women). I compared faculty salaries of five full professors chosen at random from the biology department at UNC-CH and biological sciences at NCSU (three men and two women) with NCATSU. The means and standard deviations were: $183,395 ± 34,790; $161,107 ± $26,733; and $123,633.00 ± $18,260.00, respectively. No statistical test is needed to show that faculty at UNC-CH > NCSU >> NCATSU. This fact is not contingent on what faculty I choose randomly to make the comparison. Data on faculty salaries has shown that there is a great disparity by type of institution HWI versus HBCU, as well as within institution by socially define race and gender (Renzulli et al. 2006; Jayakumar et al. 2009; Johnson and Taylor 2019; Taylor and Stan 2025).

Of course, some of you may be thinking that it is only reasonable that R1 institutions pay their faculty higher salaries compared to a research-intensive university and therefore this salary differential is not an example of structural racism. This argument would be prefaced on the idea that the research produced by the scientists who hold appointments at these institutions is more valuable as evidenced by their number of grant awards, publications, patents, and the prestige of the journals that they publish in. However, this argument is incorrect and circular. We do those things as well, and I and my colleagues collaborate in externally funded research and publish papers with faculty at UNC-CH, NCSU, and Duke and other HWI R1s in those same high prestige journals. We hold our own research awards and publish papers in those same high prestige journals, but clearly not at the same level, per the strictures placed upon us by the requirements of being a professor at a HBCU. Our colleagues at those institutions would be the first to tell you that any one of us could hold an appointment at their institutions. Yet, we do what they do without the infrastructure support that they have. The real test of this idea would be if faculty from the HWI R1s could do what they are currently doing, if they had to work under the conditions we experience at HBCUs. In short, those faculty would learn what it is like to attempt to conduct scientific research while having one hand tied behind their backs and shackles on our ankles!

The faculty salary disparity has consequences for building research programs. A few years back I attempted to recruit a very talented young researcher to come to NCATSU, but NCSU won his services because they could pay him a much higher salary than we could. We also have difficulty retaining talented faculty, because at least in the past, when one of our young faculty members achieves national or international notoriety, the R1s sweep in and recruit them away from us with the offer of higher salaries, lower teaching requirements, and better research infrastructure. Thus deciding to come to, or stay at a HBCU for our most talented scholars becomes a test of the individual’s dedication to the needs of underserved communities.

The funding disparity also impacts support staff. We have fewer support staff across the board compared to UNC-CH and NCSU. This is particularly harmful with regards to administrative staff in pre- and post-awards related to research grants. We cannot keep high quality staff in our campus IT department because they are lured away to other institutions or private industry that can pay them more competitive salaries. Our offices and rest rooms are not cleaned at the rate observed at other campuses because we simply do not have the same amount of janitorial staff. The lack of investment in HBCU infrastructure is seen and felt by faculty, staff, and our students. In short, it says to them that the state of North Carolina still values the educational experience of whites more than it does of blacks.

Yet, despite this consistent underfunding, HBCUs still graduate disproportionate numbers of Black students in STEM. Presently only about 9% of Black students are enrolled at HBCUs (down from about 18%) in 1976. However, 25% of Blacks with baccalaureate STEM degrees earned them at a HBCU. Also 30% of Blacks earning a STEM PhD received their undergraduate degree at a HBCU. From 1995 to 2004, 46% of the STEM PhDs earned by Black women, came from those who earned their undergraduate degree from a HBCU. At present, my institution NCATSU graduates more Blacks with engineering degrees than at other university in America, and also graduates more Black women with engineering degrees than any other university in America. NCATSU is ranked third in research productivity in the University of North Carolina system (UNC Chapel Hill and NCSU rank 1 and 2, respectively; University of North Carolina Research and Sponsored Programs 2023). However, we have never been allowed to have a medical school, nor did we ever receive a dollar for dollar match from the state of North Carolina for grants received from the US Department of Agriculture. We also achieve this, while teaching higher class and advising loads (to historically underserved students) and with less research infrastructure compared to our colleagues at UNC-CH and NCSU.

## The present situation

In 2004, I suggested that affirmative action programs should be “withered” in response to the rate that the United States achieved justice by socially defined race (Graves 2005b). However, affirmative action was not withered, it was abolished in July 2023 without the nation even beginning to approach social justice for its racially subordinated populations. In response to this action by the Supreme Court, I argued that the nation must invest in its HBCUs, Minority Serving Institutions (MSIs), and Tribal Colleges and Universities (Graves et al. 2023). If the nation, still values diversity in the STEM workforce, then it makes sense to invest its resources in the institutions that have been able to achieve this. With regards to diversifying the science community, HWIs have underperformed and HBCUs/MSIs/TCUs have over performed. For example, my research group alone has produced more Black women with PhDs in microbial evolution in the last 5 years, than the rest of the country has totaled in the same time period. In addition, I see changing the demography of the scientific workforce as part of an overarching reevaluation of the entire scientific enterprise (Graves et al. 2022). We need a moon-shot level investment in building the research and teaching infrastructure of the HBCUs/MSI/TCUs. The Century Foundation report of 2023 outlined what restorative justice for HBCUs should look like with regard to the USDA. This is included the following: provide $600 million in new mandatory, equity funding for 1890 institutions; incentivize states to eliminate funding inequities by phasing out the waiver for one-to-one state matching of federal research and extension formula funds to 1890 institutions; double the minimum funding percentages for appropriations for 1890 research and extension programs; provide $100 million to expand student scholarships at 1890 institutions; substantially increase other federal resources for 1890 institutions; strengthen USDA reporting to Congress on federal funding, state matching funding and state waivers granted for 1890 education, research, and extension programs. While these recommendations were specific to the USDA, the spirt of these recommendations could be adapted for other federal STEM funding agencies such as NIH and NSF.

However, the current Presidential administration, clearly does not support such bold initiatives to address historical inequities in STEM funding for HBCUs or addressing the lack of racial diversity in STEM community in general. On Tuesday January 21, 2025, President Trump signed an executive order that targeted diversity, equity, and inclusion. This order eliminated DEI programs in the federal government, and placed those who work in them on administrative leave. Some were laid off and some were transferred to other offices. In March 2026, these employees filed a complaint against their unjust termination before the United States of America Merit Systems protection board (Contreras 2025). Furthermore, as of January 23, 2025 the President temporarily suspended scientific review at the National Institutes of Health, the National Science Education, and other scientific funding agencies. Program officers at these agencies were silenced and could not respond to any questions from their constituents (Kozlov 2025.). It is clear that this silencing is part of an attempt to review the federal government spending to determine whether it is in line with the President’s agenda to eliminate: “advancing Marxist equity, transgenderism, and green new deal social engineering policies” (Vaeth 2025). On May 5, 2025, Nature reported that the National Science Foundation had stopped awarding new grants and funding existing ones. NSF staff were required to review grants and proposals for compliance with the new administration’s priorities (Garisto 2025). Several NSF program officers resigned in response to this administrative overreach.

In addition, the Trump team has deleted webpages that refer to the NIH’s programs related to DEI training programs and research. The US Federal Drug Agency pages calling for companies to conduct clinical trials on drugs and devices in diverse populations have also been deleted (Sheenhuysen 2025). Thus, it is likely that the new administration plans to cancel or greatly alter programs that primarily fund research development at HBCUs, MSIs, Hispanic Serving Institutions (HSIs), and Tribal Colleges and Universities. There is already evidence that this is underway. On March 27, President Donald Trump issued an Executive Order entitled: “Restoring Truth and Sanity to American History.” The purported goal of this action was to prevent “replacing objective facts with a distorted narrative driven by ideology rather than truth.” The order specifically criticized the Smithsonian American Art Museum exhibit “The Shape of Power: Stories of Race and American Sculpture.” The exhibit examines how “sculpture has been a powerful tool in promoting scientific racism” and “promotes the view that race is not a biological reality but a social construct.” The Trump administration considers these features of the exhibit to be a part of a “divisive, race-centered ideology.” However, this claim goes counter to over 50 years of scholarship demonstrating the non-existence of biological races in humans (Montagu 1942; Smedley 1993; Graves and Goodman 2022).

Yet, in the midst of these actions which are degrading diversity efforts, the White House issued an Executive Order on April 23, 2025 to promote excellence and innovation at Historically Black Colleges and Universities. The order actually contains several recommendations that I and my colleagues have recommended in the past, including increasing the private sector role’s in supporting HBCUs, upgrading their research infrastructure, increasing public/private sector partnerships to promote academic research and program excellence, and most importantly calling on states to promote the required state funds to 1890-land grant institutions (Graves 2005a, Graves et al. 2023). Thus far, we do not know how this initiative will be rolled out, but given the administration’s record this looks more like window dressing than substance. It is interesting to note, that while Trump was fast to threaten the elite HWIs with loss of federal funding if they did not dismantle DEI or deal with accusations of anti-Semitism, no such threats have been issued to the former confederate states that historically and continue to violate the 1890 Morrill Land Grant act.

On May 9, 2025, the administration eliminated the Division of Equity Excellence in STEM (Begin 2025). This division was charged with promoting “diversity, equity and inclusion” by removing barriers and supporting the full participation of underrepresented groups in science and engineering fields. The division’s webpage was removed from the NSF website on May 9. On that same date, all 37 divisions of the NSF were eliminated, and many of the programs within them were abolished (Mervis 2025). This is part of Trump’s plan to reduce the budget of the NSF by 55% for fiscal year 2026. During the last few weeks of April, over 1400 grants were terminated, worth over 1 billion dollars. It is feared that the drastically reduced agency will also add a new layer of review. This will make the agency more vulnerable to pressure from the White House to only fund research that suits Trump’s ideological agenda.

On May 13, 2025, Dr Alondra Nelson posted her resignation from the National Science Foundation and Library of Congress (Nelson 2025). Dr Nelson holds the Harold F. Linder chair of Social Studies at the Institute for Advanced Studies at Princeton University. She is a former CEO of the Social Science Research Council, and from 2021 to 2023 she was the Principal Deputy Director for Science and Society in the White House Office of Science and Technology Policy (OSTP). Dr Nelson was the first non-white woman and African American to lead the OSTP. She explained her resignation from the NSF and Library of Congress to Time magazine as being driven by a need to resist an administration that has no interest in honest council, uses procedural circumvention to achieve its aims, and that is actively eroding scientific freedom and replacing it with political control. We shall need more acts of courage such as this, if the scientific enterprise in America expects to survive.

While Donald Trump claims to not support the Project 2025 document, thus far he has followed many of its suggestions as seen by the examples above (Greene 2024). One of the most egregious of its suggestions is the plan to dismantle the Department of Education. If the Department of Education is eliminated, programs such as Title III, which helps institutions of higher education become self-sufficient and expand their capacity to serve low-income students would be endangered. Many of our graduate students at NCATSU have been able to complete their STEM PhD degrees due to Title III funding. In the place of the Department Education Project 2025 proposes to give block grants to support education directly to the states. Should this occur, it will be difficult to predict how they will respond toward their HBCUs, MSI, HSIs, and TCUs. For the most part, state legislatures in the former Confederate states share the same racial ideologies as the incoming president. So there will be no help from them either. I suspect that their attitude toward the HBCUs/MSIs/TCUs will be to allow them to wither on wine.

So far, private industry has not fully weighed in on their plans regarding DEI. Some such as Amazon, Target, and Walmart plan to eliminate their DEI programs. Others such as COSTCO still recognize the value of diversity in their workforce (Cavale 2025). Therefore, these current events need to be a clarion call for those who have the financial means to invest heavily in improving the infrastructure of HBCUs/MSIs/TCUs. At present, there are over 800 billionaires in the United States, who hold in excess of 6.22 trillion dollars in wealth (Collins 2024). If even one-tenth of this number (and some already have) were to decide to engage in social philanthropy dedicated to these institutions, great progress could be made within the decade. Even without the commitment of billionaires, citizens can (and should) donate in greater numbers to these institutions. Furthermore, we should be holding the legislatures of the former Confederate states accountable for their history of underfunding HBCUs. In 2021, the four HBCUs in Maryland settled a federal law suit calling for the state to pay the funds withheld from them at the amount of 577 million dollars over the decade (Shwe 2021). As the underfunding of HBCUs has occurred in every state, students, student’s families, professional societies, and the public at large should be bringing these class action suites to provide restorative justice.

## A call to action

Under the present circumstances, the field and function of the HBCUs, MSIs, HSIs, and Tribal Colleges and Universities have not changed. But their role in American society has never been more important. The organized attack on racial justice by eliminating efforts to promote DEI in American society has compromised the capacity for HWIs to adequately address this issue. This is because these institutions are at war with themselves over what DEI means and how, if at all, they can implement programs to achieve it (Gutkin 2025). Most of the DEI programs in the states that voted for Trump in 2024 have already been eliminated in response to the threat that they would lose their federal funding. As of April 30, 2025, 21 states, the District of Columbia, and Puerto Rico have certified that they no longer use DEI in higher education (Stone and Lieberman 2025). Even private institutions for fear of losing federal funding have dismantled or renamed DEI programs. However, close to 400 universities and colleges have refused to comply with the order (McCarthy 2025).

No such confusion exists about the meaning of DEI nor how to achieve it in the HBCUs. DEI means providing an equality of opportunity for individuals without regard to their socially defined race, gender, or sexual orientation. DEI is not in opposition to merit, but is an essential component of merit criteria (and vice versa). These programs are necessary to redress historical and ongoing structural racism within American society. Removing the structural racist barriers preventing individuals from entering STEM careers will improve the quality of the scientific enterprise (Graves et al. 2022). It matters who is in the room when scientific research programs are conceived and implemented. The example of the Trump administration forcing the FDA to take down webpages calling for diversity in clinical trials powerfully makes this point. It is not political correctness or woke ideology that supports the need for a greater diversity of individuals in clinical trials. This is required because the more genetic diversity in the trial the greater the likelihood that idiosyncratic and harmful type 2 drug reactions will be discovered. No one should be opposed to the manufacture of drugs that work well for everyone.

Under the current circumstances, HBCU and other MSIs hold the key to changing the demography of the scientific workforce. We have the know-how, and we have faculty who are dedicated to producing top quality research as well as top quality students. What we lack is a level playing field. Indeed, the current attacks on the NSF, NIH, EPA, NOAA, and other federal scientific funding agencies has made the field even more uneven. With an even smaller amount of funds to disperse, institutions that have more research infrastructure (faculty, instrumentation, and support staff) are far more likely to win in competition against institutions with much less of those resources. In addition to that disadvantage, the potential of adding an additional ideological level of review that will require research awards to be in concert with the administration’s agenda will also further harm HBCUs, HSIs, and Tribal Universities and Colleges. For example, health disparity research is often conducted at MSIs, and this topic is a non-starter in the Trump administration.

At present, only one HBCU (Howard University) holds the distinction of being a Carnegie research-1 institution (Weissman 2024). NCATSU missed R1 status in 2024 by only two PhD students per year. However, with many of our NSF/NIH research and training programs supporting graduate students being canceled, we are now scrambling to find ways to support our students. The underdevelopment of the HBCU research enterprise is a testament to the cumulative impacts of structural racism in American society.

The reader may ask what can they do to help eliminate structural racism in science if they are a faculty member at an HWI. First, it requires that you recognize that this problem exists. I spent the beginning of my career at R1 HWIs. Most of the STEM faculty that I interacted with in these institutions were clueless to this issue. Some were actively hostile to efforts to redress structural racism in science. Fewer were committed to doing the work to help it eliminate it. From what I can observe, this situation has not changed much over the years. More than ever, this is not something that an individual can achieve alone. It requires networking with like-minded individuals. It requires doing the work to learn about the dynamics of structural racism. You do not have to re-invent the wheel as excellent resources exist on this problem (see for example, the Racial Equity Institute, https://racialequityinstitute.org/). It requires forming alliances with other interest groups. The dynamics of racism, sexism, and anti-LGBTQ + bigotry share many features in common. Too often progress has not been made due to socially oppressed groups fighting for scraps from the master’s table.

Finally, the question must be addressed as what is to be gained by eliminating structural racism in science. For example, why should a person from a socially advantaged group join in the struggle to eliminate such as advantages. Again, I argue that inclusion and equity will make the scientific enterprise better (Barabino et al. 2026). In this paper, we outlined a series of actions that can lead to a sustainable change in the scientific enterprise. These actions must be implemented in pre-college preparation, scientific workforce culture, and to progress science to meet the needs of all people. This starts with scientists becoming educated about the role that scientific enterprise has played (and is still playing) in the oppression of people. We must consider how we can become engaged in socially responsible science. This is precisely the approach we are taking in our NSF-funded Engineering Research Center for Precision Microbiome Engineering (PreMiEr: https://premier-microbiome.org/societal-ethical-implications/). The societal and cultural implications of our research is a core pillar in our center and not an afterthought. We are also engaged in pre-college education by helping to empower K-12 teachers to incorporate cutting edge biomedical research via the North Carolina Amgen Biotechnology program (https://amgenbiotechexperience.net/northcarolina/). We are providing this training free of charge to teachers across North Carolina, mainly in majority minority school districts with approximately 50% of students eligible for free and reduced lunch. Also, we are directly engaged in changing the scientific workforce culture. Our senior biology faculty take a direct role in mentoring our junior faculty, but also supporting them by speaking out against all forms of harassment in our university and scientific societies. We also model servant leadership, via things such as tiered mentorship of not just our junior faculty, but also of their postdoctoral, graduate student, and undergraduate researchers.

However, HBCUs, MSIs, and other under resourced colleges and universities cannot achieve these changes by themselves. Well-resourced HWIs must learn how to form honest research collaborations with under resourced universities. At NCATSU, we are routinely approached by R1 institutions who simply want to recruit our students to come to their campuses to meet their DEI needs. We no longer accept such one-sided relationships. Some of our most successful and balanced research collaborations include the NSF-funded Science Technology Center Biocomputational Evolution in Action (BEACON) that included Michigan State University; University of Idaho; University of Texas, Austin; and the University of Washington (2014–2024; Banzhof et al. 2020) and our current ERC PreMiEr. In the summer of 2024, we began a research and training collaboration with colleagues at UC Santa Cruz in which students from UC Santa Cruz came to NCATSU, and this summer students from NCATSU will spend time at UC Santa Cruz.

Improving our culture of science will improve its capacity to address some our most vexing and existential problems (Graves 2022; Graves et al. 2022). This will require us to always think deeply about how the scientific enterprise and the technological innovations it produces impact multiple stake holders, particularly those that have been historically marginalized in the society of science. This will also build greater trust among society as a whole concerning the scientific enterprise. To achieve this, we need build a meaningful and two-way exchange with communities that have been and are continually marginalized in our society. I suggest that if we had achieved that we would not have been as vulnerable to the recent dismantling of federally funded scientific research.

The alternative, to changing the agenda of science on the other hand, is barbarism. We have a president who does not accept the science of climate change, and who has appointed a head of Health and Human Services who does not accept the science of vaccination. The scientific enterprise is never separated from social issues. Thus it is time for American scientists to think more deeply about what kind of society that they want to do their science in and for. However, intellectual activity divorced from action is useless. American scientists both as individuals and professional organizations need to let the nation know how the current administration’s views are harming the scientific enterprise, and the consequences of such reckless action. Dr Alondra Nelson’s example is a good place to start, but we need more prominent scientists and organizations to publicly stand up to the Trump administration. Informing the nation about the critical role of scientific research and how dismantling it will harm our future can be done by statements to the press, by speaking to community organizations, and by aligning with other organization dedicated to societal good (Graves 2025b).

Finally, this will require the willingness to take non-violent action against this administration’s policies. These could include days of action, where scientists refuse to teach classes or conduct research. We can organize teach-ins to help the public better understand why a healthy scientific research enterprise benefits our society. We should consider organizing a “science march on Washington.” On April 17, 2025, more than 150 campuses took part in a day of action to protest against Trump’s assaults on higher education (Conley 2025). Clearly, while this was a good start, we will need broader public efforts to thwart this administration’s anti-science agenda. Whenever possible we should be writing opinion pieces for local and national newspapers to raise consciousness around the costs of dismantling our scientific infrastructure. In person meetings with local, state, and national legislators can be organized by scientific organizations.

## Conclusion

This paper has outlined the history of HBCUs. It has recounted the obstacles they faced due to historic and ongoing structural racism in American society. Despite these obstacles, HBCUs have achieved important outcomes, including producing differential numbers of undergraduates in STEM. Yet, this achievement has not been sufficient to eliminate structural racism in US society. However, HBCUs have an important role to play in that regard. To move toward this goal, we require equitable funding. The underfunding of HBCUs in the former confederate states is a violation of the law. Given the present environment this situation is unlikely to get better. Thus, to overcome this obstacle, we require real allies in the HWI institutions. Together we can actualize a new agenda for scientific research that is inclusive and responsive to long neglected societal needs. Let us remember that the student sit-ins that catalyzed the integration of American society began with four North Carolina A&T students who sat down at the Woolworth’s lunch counter in Greensboro, NC; and that the last leader of America’s most important movement toward racial justice graduated from Morehouse College.
